# The Safe Start trial to assess the effect of an infant hygiene intervention on enteric infections and diarrhoea in low-income informal neighbourhoods of Kisumu, Kenya: a study protocol for a cluster randomized controlled trial

**DOI:** 10.1186/s12879-019-4657-0

**Published:** 2019-12-19

**Authors:** Jane Mumma, Sheillah Simiyu, Evalyne Aseyo, John Anderson, Alexandra Czerniewska, Elizabeth Allen, Robert Dreibelbis, Kelly K. Baker, Oliver Cumming

**Affiliations:** 1 Center of Research, Great Lakes University Kisumu, P.O. Box 2224-40100, Kisumu, Kenya; 2 Urbanisation and Well Being Unit, African Population and Health Research Center, P.O. Box 10787-00100, Nairobi, Kenya; 3 Independent Research Consultant, TX78702, Austin, USA; 4 Department of Medical Statistics, London School of Hygiene and Tropical Medicine, WC1E 7HT, London, UK; 5 Disease Control Department, London School of Hygiene and Tropical Medicine, WC1E 7HT, London, UK; 6 Department of Occupational and Environmental Health College of Public Health, University of Iowa, Iowa City, IA 52333 USA

**Keywords:** Enteric infections, Diarrhoea, Child food, Infant food, Hygiene, Kenya, Kisumu

## Abstract

**Background:**

*Symptomatic and asymptomatic enteric infections* in early childhood are associated with negative effects on childhood growth and development, especially in low and middle-income countries, and food may be an important transmission route. Although basic food hygiene practices might reduce exposure to faecal pathogens and resulting infections, there have been few rigorous interventions studies to assess this, and no studies in low income urban settings where risks are plausibly very high. The aim of this study is to evaluate the impact of a novel infant food hygiene intervention on infant enteric infections and diarrhoea in peri-urban settlements of Kisumu, Kenya.

**Methods:**

This is a cluster randomized control trial with 50 clusters, representing the catchment areas of Community Health Volunteers (CHVs), randomly assigned to intervention or control, and a total of 750 infants recruited on a rolling basis at 22 weeks of age and then followed for 15 weeks. The intervention targeted four key caregiver behaviours related to food hygiene: 1) hand washing with soap before infant food preparation and feeding; 2) bringing all infant food to the boil before feeding, including when reheating or reserving; 3) storing all infant food in sealed containers; and, 4) using only specific utensils for infant feeding which are kept separate and clean.

**Results:**

The primary outcome of interest is the prevalence of one or more of 23 pre-specified enteric infections, determined using quantitative real-time polymerase chain reaction for enteric pathogen gene targets. In addition, infant food samples were collected at 33 weeks, and faecal indicator bacteria (*Enterococcus*) isolated and enumerated to assess the impact of the intervention on infant food contamination.

**Conclusion:**

To our knowledge this is the first randomized controlled trial to assess the effect of an infant food hygiene intervention on enteric infections in a high burden, low income urban setting. Our trial responds to growing evidence that food may be a key pathway for early childhood enteric infection and disease and that basic food hygiene behaviours may be able to mitigate these risks. The Safe Start trial seeks to provide new evidence as to whether a locally appropriate infant food hygiene intervention delivered through the local health extension system can improve the health of young children.

**Trial registration:**

The trial was registered at clinicaltrial.gov on March 16th 2018 before enrolment of any participants (https://clinicaltrials.gov/ct2/show/NCT03468114).

## Background

Diarrhoeal disease, a key symptom of gastro-intestinal or enteric infection, is the fourth leading cause of disability globally [1] and the leading cause of child death in sub-Saharan Africa [2]. Furthermore, there is growing evidence of the impact of sub-clinical childhood enteric infection and disease on growth and development [3, 4].

Food is likely to be an important source of exposure to enteric pathogens in early childhood. Recent studies have shown that food given to children in early childhood can be highly contaminated with faecal indicator bacteria [5] as well as specific diarrhoeagenic enteric pathogens [6]. Environmental interventions to reduce exposure to these pathogens and reduce diarrhoea have traditionally focused on improving the quality and distribution of drinking water, the management of excreta through sanitation systems and the promotion of handwashing with soap at critical times [7] but generally not on food hygiene related behaviours and infrastructure.

More than half of the world’s population now reside in urban areas and over one third of this population live in ‘slums or informal settlements’ [8]. Although access to safe water and sanitation is generally higher in urban areas [9], the risk of enteric infection may be greatest in poor urban areas due to the combination of high population density and limited public health infrastructure [10–13]. These conditions pose multiple risks for contamination of food as supported by a recent study of pathogen diversity in infant food in low-income informal neighbourhoods of Kisumu, Kenya [6]. The ‘Safe Start’ trial is designed to assess whether a locally appropriate, low-cost food hygiene intervention, delivered within the context of the existing health extension system in peri-urban neighbourhoods of Kisumu, Kenya can reduce early childhood exposure to enteric pathogens.

## Methods

### Research aim and objectives

The purpose of this study is to examine the effect of an infant food hygiene behaviour change intervention on child health. The study will assess the impact of the intervention on: (1) infant health as determined by prevalence of gastro-intestinal infection and diarrhoeal; (2) specific food hygiene practices; and (3) infant food contamination.

### Study design

**Fig. 1:**
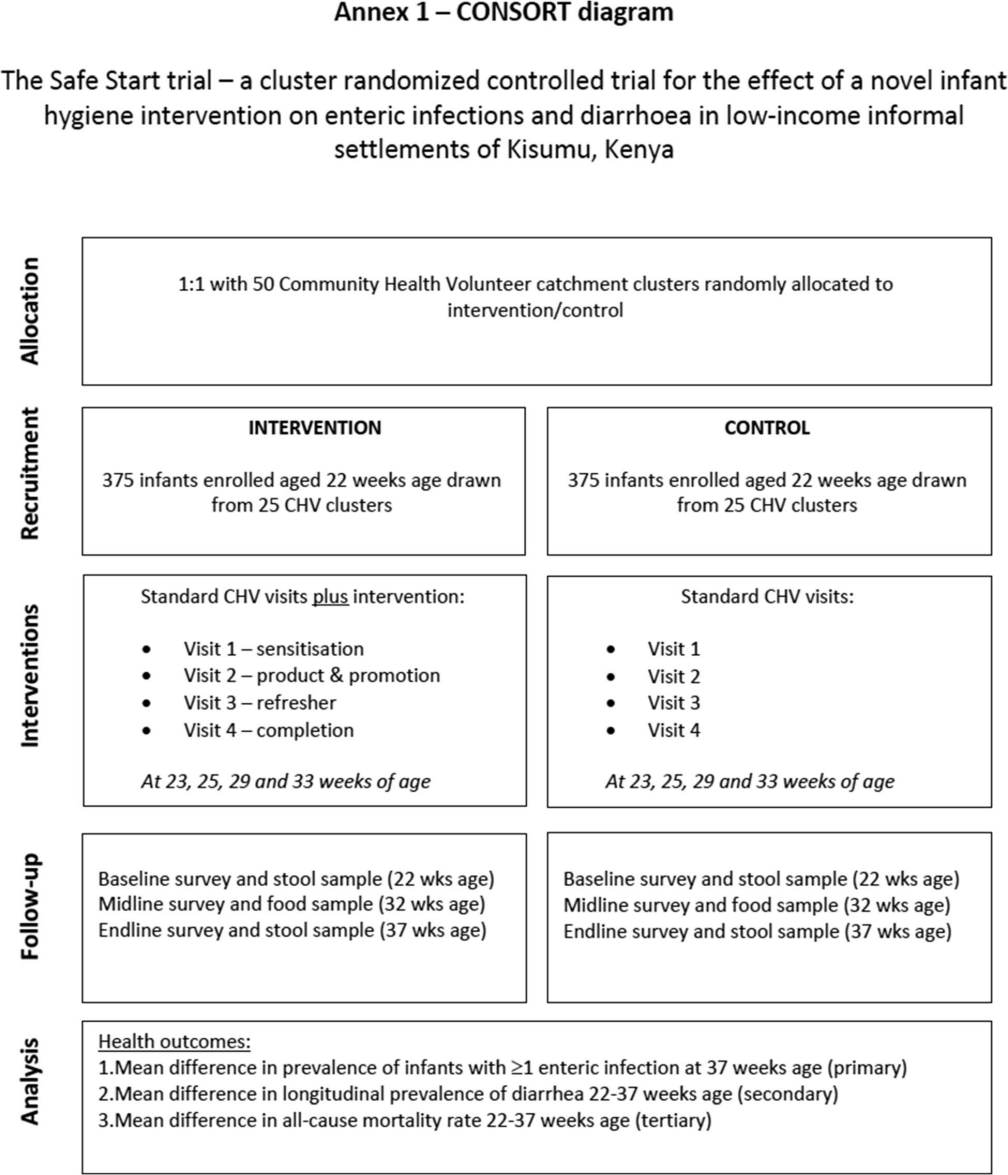
– CONSORT diagram

Our study was a cluster randomized controlled trial (cRCT) design. Clusters for the trial were defined as the catchment areas of local Community Health Volunteers (CHVs); a total of 50 CHV catchment areas were recruited into the study and randomly assigned to an intervention and control arm of the study. An overview of the study design is presented in Fig. 1 (CONSORT [14] diagram).

The primary outcome for the study is the prevalence of enteric infection at age 37 weeks (+/− 1 week). We define the prevalence of enteric infection as the presence of 1 or more enteric pathogens in child stools based on the detection of 23 genetic markers of specific common enteric bacteria, viruses and protozoan (Table 1). The secondary outcome is diarrhoea; defined as the number of days a child has diarrhoea between 22 and 37 weeks of age (+/− 1 week). Tertiary outcomes include child mortality, defined as any infant death occurring between 22 and 37 weeks of age (+/− 1 week). In addition, the study will assess the effectiveness of the intervention by measuring changes in specific food practices and in bacterial contamination of infant food.

**Table 1:** – Specific enteric pathogen primers and probes for TaqMan Array Card used to determine the primary outcome

PATHOGEN	GENE TARGET	FORWARD PRIMER	REVERSE PRIMER	PROBE SEQUENCE	Ref
BACTERIA					
Aeromonas	Aerolysin	TYCGYTACCAGTGGGACAAG	CCRGCAAACTGGCTCTCG	CAGTTCCAGTCCCACCACTT	[2]
*Campylobacter jejuni/C. coli*	*cadF*	CTGCTAAACCATAGAAATAAAATTTCTCAC	CTTTGAAGGTAATTTAGATATGGATAATCG	CATTTTGACGATTTTTGGCTTGA	[2]
*Clostridium difficile*	*tcdB*	GGTATTACCTAATGCTCCAAATAG	TTTGTGCCATCATTTTCTAAGC	CCTGGTGTCCATCCTGTTTC	[2]
Enteroaggregative *Escherichia coli* (EAEC)	*aaiC*	ATTGTCCTCAGGCATTTCAC	ACGACACCCCTGATAAACAA	TAGTGCATACTCATCATTTAAG	[2]
Enteroaggregative *Escherichia coli* (EAEC)	*aatA*	CTGGCGAAAGACTGTATCAT	TTTTGCTTCATAAGCCGATAGA	TGGTTCTCATCTATTACAGACAGC	[2]
Enterohemorrhagic *E. coli* (EHEC) 0157	*rdbE*	TTTCACACTTATTGGATGGTCTCAA	CGATGAGTTTATCTGCAAGGTGAT	CTCTCTTTCCTCTGCGGTCCT	[1]
Enteropathogenic *E. coli (*EPEC)	*eae*	CATTGATCAGGATTTTTCTGGTGATA	CTCATGCGGAAATAGCCGTTA	ATACTGGCGAGACTATTTCAA	[2]
Enteropathogenic *E. coli (*EPEC)	*bfpA*	TGGTGCTTGCGCTTGCT	CGTTGCGCTCATTACTTCTG	CAGTCTGCGTCTGATTCCAA	[2]
Enterotoxigenic *E. coli* (ETEC) LT toxin	ETEC LT	TTCCCACCGGATCACCAA	CAACCTTGTGGTGCATGATGA	CTTGGAGAGAAGAACCCT	[2]
Enterotoxigenic *E. coli* (ETEC) ST toxin	STh STp	GCTAAACCAGYAGRGTCTTCAAAATGAATCACTTGACTCTTCAAAA	CCCGGTACARGCAGGATTACAACATGAATCACTTGACTCTTCAAAA	TGGTCCTGAAAGCATGAATGAACAACACATTTTACTGCT	[2]
*Salmonella enteritidis*	*ttr*	CTCACCAGGAGATTACAACATGG	AGCTCAGACCAAAAGTGACCATC	CACCGACGGCGAGACCGACTTT	[2]
*Shigella* spp.	*virG*	TCAGAAAGGTAATTGGCATGGA	AGAACCGCGCCCAAAGA	AGGGCGGAATATT	[1]
*Vibrio cholerae*	*hlyA*	ATCGTCAGTTTGGAGCCAGT	TCGATGCGTTAAACACGAAG	ACCGATGCGATTGCCCAA	[2]
PROCESS CONTROL					
MS2	MS2g1	TGGCACTACCCCTCTCCGTATTCAC	GTACGGGCGACCCCACGATGAC	CACATCGATAGATCAAGGTGCCTACAAGC	[2]
VIRUS					
Adenovirus 40–41	Fiber Gene	AACTTTCTCTCTTAATAGACGCC	AGGGGGCTAGAAAACAAAA	CTGACACGGGCACTCT	[2]
Adenovirus broad species	Hexon	GCCACGGTGGGGTTTCTAAACTT	GCCCCAGTGGTCTTACATGCACATC	TGCACCAGACCCGGGCTCAG	[1]
Norovirus GI	ORF 1–2	CGYTGGATGCGNTTYCATGA	CTTAGACGCCATCATCATTYAC	TGGACAGGAGATCGC	[1]
Norovirus GII	ORF 1–2	CARGARBCNATGTTYAGR TGGATGAG	TCGACGCCATCTTCATTCACA	TGGGAGGGCGATCGCAATCT	[2]
Rotavirus	NSP3	ACCATCTWCACRTRACCCTCTATGAG	GGTCACATAACGCCCCTATAGC	AGTTAAAAGCTAACACTGTCAAA	[2]
PROTOZOAN					
*Giardia duodenalis* Assemblage A	*triosephosphate isomerase (TPI)*	TTCCGCCGTACACCTGTC	GCGCTGCTATCCTCAACTG	ATTGCGGCAAACACGTCA	[1]
*Giardia duodenalis* Assemblage B	*triosephosphate isomerase (TPI)*	GATGAACGCAAGGCCAATAA	CTTTGATTCTCCAATCTCCTTCTT	AATATTGCTCAGCTCGAGGC	[1]
*Cryptosporidium spp*.	18 s rRNA	GGGTTGTATTTATTAGATAAAGAACCA	AGGCCAATACCCTACCGTCT	TGACATATCATTCAAGTTTCTGAC	[2]
*C. hominus*	LIB13	TCCTTGAAATGAATATTTGTGACTCG	AAATGTGGTAGTTGCGGTTGAAA	CTTACTTCGTGGCGGCGT	[1]
*C. parvum*	LIB13	TCCTTGAAATGAATATTTGTGACTCG	TTAATGTGGTAGTTGCGGTTGAAC	TATCTCTTCGTAGCGGCGTA	[1]

### Study setting

**Fig. 2:**
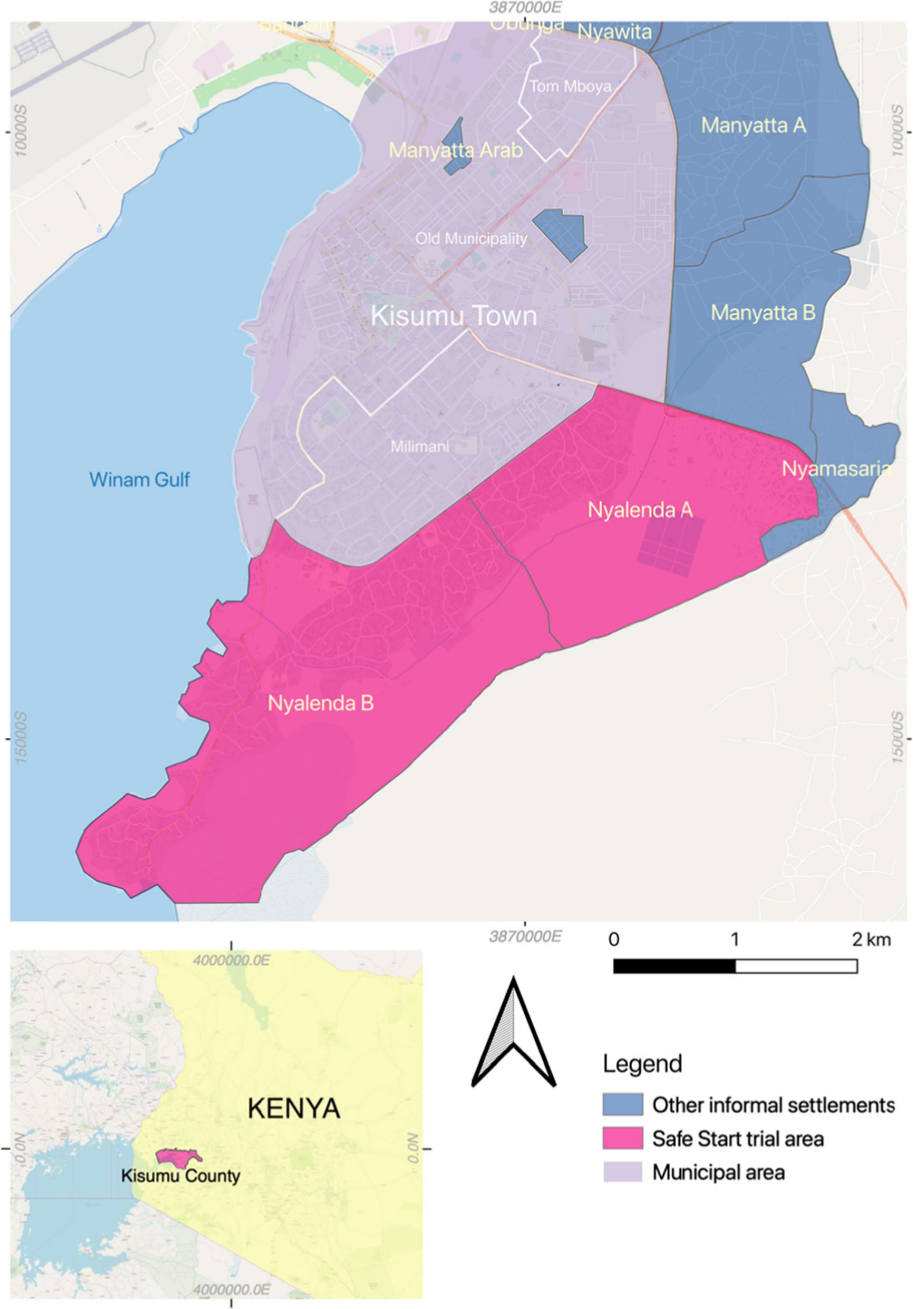
– Map showing Safe Start study areas of Nyalenda A and B (pink), two of the informal neighbourhoods around Kisumu Town in Kisumu County, Kenya

The study is being conducted in two informal neighbourhoods of Kisumu, Kenya: Nyalenda A and Nyalenda B (Fig. 2). Kisumu is the third largest city in Kenya and is located in Kisumu County, on the shores of Lake Victoria, and has a population of approximately 400,000. The city is surrounded by a series of peri-urban areas sometimes referred to as the ‘slum belt’ [15]. These peri-urban areas have emerged due to economic migration and a lack of affordable housing [16]. Some sources estimate that up to 60% of the city’s population reside in these peri-urban communities [17].

The counties that previously made up the Nyanza and Western provinces have relatively high levels of infectious disease morbidity and mortality. The child mortality rate for Kisumu county is 105 deaths per 1000 live births and the prevalence of childhood stunting (below-2 SD) is approximately 25% [18]. In Kisumu county, approximately 70% of all children between 12 and 23 months of age have received all recommended child disease vaccines, and it is estimated that 30% of children experiencing diarrhoea receive timely oral rehydration therapy [ORT] [18]. Two-week diarrhoeal prevalence in Kisumu is 18%, higher than neighbouring areas [18]. Data from the nearby Kenyan site of the Global Enteric Multi-site Study (GEMS) [19] reported the leading identified infectious causes of diarhhoea to be Rotavirus, *Cryptosporidium*, ST-ETEC and *Shigella*.

### Community health volunteer system

Kenya has been undergoing a process of decentralization, with many areas of policy, including the health sector and the community extension services, now the responsibility of the County Government. The Community Health Committee (CHC), is the health governance structure closest to the people at the county level. Community Health Volunteers (CHVs), who serve as frontline health workers in this decentralized system, report to the community health committee through the Community Health Extension Workers (CHEW) [20].

CHVs receive basic training to diagnose and treat illnesses such as malaria, pneumonia, and diarrhoea; make referrals to health facilities; provide health education; conduct nutrition surveillance; collect vital events data; assist with immunization and provide other aspects of maternal and child health [21, 22]. More recently, CHVs have been engaged in the promotion of some hygiene-related behaviour, including community led total sanitation (CLTS) and safe household water storage and treatment [23]. Under the current study, we collaborated with CHVs to design and test an intervention to reduce infant exposure to enteric pathogens and they are involved in the delivery of the intervention.

### Study participants

Our primary participants are infants enrolled at the age of 22 weeks (+/− 1 week), who currently reside in Nyalenda A or B, and will be living there for the subsequent five months. Our secondary participants are primary or secondary caregivers who provide care to the infant during the day and who are at least 18 years of age. A primary caregiver is defined as the person who is directly responsible for the enrolled child and a secondary caregiver is defined as any other person apart from the primary caregiver who watches the child or supports the primary caregiver.

### The Safe Start intervention

#### Development of intervention

We followed the Behaviour Centered Design (BCD) approach to intervention development [24]. Specific qualitative and quantitative formative research studies were implemented in a similar and neighbouring area of Kisumu city. Infant faecal-oral exposure in their domestic environment was assessed using structured observation of infants and caregivers, identifying low rates of hand hygiene among caretakers and infant food as a viable route of exposure to enteric pathogens that could be mitigated by safe preparation, storage and reheating of food [25]. Caregiver attitudes and practices in this population and the emotional and environmental drivers of food hygiene behaviours were assessed through structured observation and in-depth interviews with primary and secondary caregivers [26]. Microbiological and molecular analysis of infant food samples was used to determine the prevalence and intensity of infant food contamination with specific enteric pathogens implicated in childhood diarrhoea [6]. Various known diarrhoeagenic agents, including bacteria, viruses and protozoa, were frequently detected with at least one enteric pathogen identified in 62% of infant food samples and multiple pathogens identified in 37% of infant food. A fourth study that specifically informed Safe Start intervention delivery explored CHV schedules, routines and capacity to deliver behaviour change through direct observation, interviews, and focus group discussions. This study identified a wide range of challenges, including: poor training, lack of material resources, and limited incentives to undertake additional tasks [23].

Formative research findings led to the design of two primary candidate intervention components designed to improve food hygiene behaviours in the target population. The first component consisted of hardware items introduced at the household level to facilitate improved food hygiene behaviours. The second component consisted of motivational and educational messaging designed to improve caregiver knowledge of proper food hygiene and target the specific emotional drivers of safe food hygiene identified in formative research. The feasibility and acceptability of the two intervention components – both independently and in combination– were assessed and iteratively adapted using the Trials of Improved Practice (TIPs) methodology [27]. Details of this process are described in Simiyu et al. [28].

#### Intervention description

The final intervention was designed to target early childhood exposure to enteric pathogens through contaminated food. The intervention targets the following four behaviours:

1. Safe hand hygiene: handwashing with soap before food preparation and before infant feeding.
2. Safe food preparation: bringing all infant food to the boil before any feeding event.
3. Safe storage of food: storing all infant food in sealed containers.
4. Safe feeding: using designated utensils for infant feeding reserved from other use.

The intervention components use two sequential and complementary aspects of the nurture motives. The first is the desire to care for and protect a child as they grow. In formative research, “happy” was seen as marker of child fitness and health. The concept of “Happy Baby” emerged as a focal point for messaging and was incorporated into intervention materials. The second commonly articulated aspect of nurture was the desire to ensure that the child will have a successful future. This was operationalized as messages related to a “Successful Child” and focused on ensuring that the mother provides the necessary foundation for future success. In addition to messages targeting emotional drivers, the intervention also provides the necessary foundational knowledge about food hygiene, and associated risks, but framed within an emic understanding of child health and successful parenting within the communities.

**Fig. 3:**
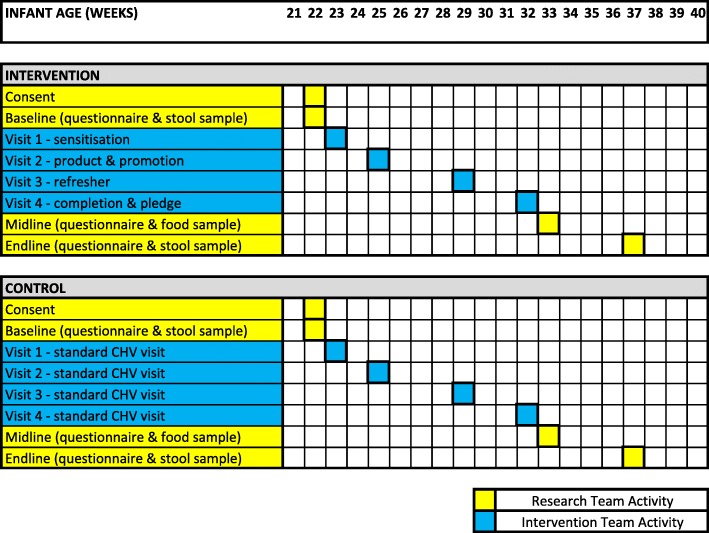
Intervention and Data Collection Schedule

The intervention is delivered in four visits (Fig. 3) in collaboration between CHVs and specifically trained field staff. Visit 1 is a preliminary sensitization visit, led by participating CHVs in the weeks before children turn six months of age. During this first visit, CHVs reiterate existing messages regarding the importance of exclusive breastfeeding until 6 months of age, appropriate weaning foods, and their introduction after six months. The CHV also introduces new topics regarding food hygiene, including: environmental contamination, the risks associated with contaminated weaning food, and the potential health consequences - diarrhoeal disease, growth impairment, and cognitive deficits. The second visit is timed to coincide with children turning 6 months (25 weeks) old and introduces the “Happy Baby” aspect of the intervention. This household visit is designed to be a fun and lively experience for participating households and is led by specifically trained field staff who are accompanied by local CHVs. During this visit, field staff deliver a number of products designed to enable and trigger improved food hygiene practices, including: a baby bowl, a baby spoon, a baby cup, a handwashing container/station, a bottle dispenser of liquid soap (with instructions for self-refill), two deep and two rectangular sealable storage containers, and a branded “Happy Baby” feeding mat. In addition, intervention households receive a “Happy Baby” customised calendar with images that reinforce target behaviours and reference newly provided materials. Caregivers are instructed to record diarrhoea episodes on calendars between visits, ensuring that caretakers interact with and see messages. Visit 3 occurs when the child is 29 weeks old. This visit, once again lead by local CHVs, reinforces messages, discusses experiences with new target behaviours, and reviews new information on food hygiene. Visit 4 occurs when the child is 32 weeks old and introduces the “Successful Child” component of the intervention. Successful child images compliment “Happy Baby” materials by including images of older children in graduation gowns and caps. The successful child stage includes a “graduation event” for the caregiver, including a “food hygiene pledge”, and a forward-looking discussion about their aspirations for the infant and how to give their child a “Safe Start” in life. As an example of the materials, we include an image of the “Successful Girl” calendar given to caregivers in the intervention group (Additional file 1).

#### Data collection

Data are collected at three points – baseline, midline, and endline – through survey questionnaire, structured observation, along with stool and food sample collection (Fig. 3). At baseline (22 weeks of age), a short survey questionnaire is administered to the infant caregiver covering general household information, WASH access, infant health and animal contacts, with key details verified against the infant’s health card (e.g. date and place of birth, vaccination status). At the same time, a stool sample is collected from the infant for analysis (procedure described below). At midline (33 weeks of age) a second household visit is made with a structured observation of infant food preparation and feeding by the caregiver, and a second short questionnaire administered. Lastly, an endline visit is completed at age 37 weeks when a stool sample is collected and a third short questionnaire administered.

Intervention ‘fidelity’ is assessed using process evaluation methods [29] to collect qualitative and quantitative data through in-depth interviews, focus group discussions and structured questionnaires with CHVs and caregivers among a small sample of intervention and control clusters/households. At each follow-up point, any participant deaths are recorded along with the official cause of death.

All personal identifiers collected, including names and telephone numbers, will be stored separately from other, de-identified data. All data from the surveys, stool and environmental samples will be linked through a unique household code that cannot be traced back to an individual. GPS coordinates for individual households will recorded which represents identifying data that therefore requires careful protection. The GPS coordinates themselves, and the specific locations of households on maps, will not be published or presented with results of any analyses. All physical forms will be kept in a locked file cabinet in a locked office to prevent unintended release of information. All electronic data will be encrypted and stored on secured and password protected electronic databases.

#### Environmental and clinical sample collection

A stool sample is collected for each enrolled infant at baseline (22 weeks of age) and endline (37 weeks of age), and an infant food sample collected at midline (33 weeks of age) [Fig. 3]. For infant stool, the infant’s caregiver is given several unused, clean diapers and is asked to use the diapers on the child until they defecate. Once a child has defecated in a diaper, the caregiver folds the diaper so that the faeces is undisturbed on the interior and places the diaper in a provided biohazard bag. This procedure is used to prevent faeces samples from being collected off the ground (contaminated by soil) or from out of potties used by other children (contaminated by faeces). The bag is stored in a cool, dark, secure place until the research team returns to the household the next day and collects the sample. On the day of sample collection, the enumerator uses the scoop from the sterile collection bottle to scoop the stool from the diaper into the bottle, labelling the container with the date of collection and participant’s identification number. The bottle with the stool sample is placed in a bio hazard bag and the bag placed on ice in a cooler box and transported to the laboratory. At the laboratory, a lab technician sterilizes the outside of the bio hazard bag, removes the stool collection bottle from the bag, and records the sample as received. If the infant has not defecated on the day of sample collection or the stool sample is not sufficient for collection, the enumerator informs the parent or caregiver that they will return again the next day. This continues for up to 5 consecutive days.

For the infant food sample, the research team collects a sample of food cooked during the midline observation, and again several hours later after food has been used and stored for several hours. The caregiver is asked to place a sample of food in a sterile WhirlPak bag by the same means as she would feed a child (e.g. spoon, hands). Given that levels of contamination in food may increase with time during the day, time of collection is noted. Samples are labelled (date, time and study identification number), placed immediately into a cooler box, maintained at < 10 °C with ice packs, and then transported to the laboratory for analysis.

#### Laboratory analysis

Food samples are processed by enumerating a bacterial indicator of faecal contamination (*Enterococcus*). In brief, 1 ml (mL), 0.1 mL, and 0.01 mL dilutions of liquid foods are filtered through 0.45 μm pore-size membrane filter (Millipore Corp., Bedford, MA, USA), and the filters are cultured overnight on Slanetz &Barley Enterococcus Medium (OXOID CM0377). For solid foods, five grams are homogenized with 45 mL of sterile phosphate buffer saline (PBS), and 10 mL, 1 mL, and 0.1 mL dilutions are filtered and cultured on Enterococcus agar plates. Then the plates are incubated at 41 °C ± 0.5° for 24 h. After incubation, all light and dark red colonies are counted as *Enterococcus* and expressed as colony forming units (CFU) present per gram of food sample. A 10 ml volume of PBS used to resuspend solid food samples and wash membrane filters is processed each day as a food negative control.

A 200 mg sample of each stool sample is measured into a Zymo Shield Collection container and DNA and RNA is co-extracted using the ZymoBiomics DNA/RNA Mini kit according to the manufacture’s protocol (Zymo Corp., CA, USA). DNA/RNA is immediately stored in a − 20 °C freezer until transfer to the University of Iowa for molecular analysis. A second 200 mg stool sample is transferred to a labelled sterile Eppendorf tube and stored in a − 20 °C freezer as a repository in the event that primary samples are lost, mislabelled, or otherwise destroyed. All stools are processed in sterilized biosafety cabinets with laminar air flow, and one process negative control is prepared each day by leaving a Zymo Shield Tube open in the cabinet during stool processing, and then processing it for DNA/RNA extraction. Pathogen targets are detected and quantified by quantitative real-time polymerase chain reaction using Customized Taqman Array Cards on a ViiA7 thermocycler (Life Technologies, USA) as previously described with the exception of adding 300 uM bovine serum albumin (BSA) to reduce inhibition during PCR. Outcomes are defined as the pathogen-specific presence and concentration of individual pathogens, as well as the presence and diversity (sum of pathogen types) of all pathogens. Concentrations of individual pathogens per gram of stool are estimated by comparison of cycle thresholds of pathogen specific genes against standard curves for each reference of interest. In the event that pathogen genes are detected in process negative controls, monoplex PCR is used to verify that detection is true contamination. If negative controls are contaminated, the stool samples processed on the same day as the negative control are considered non-determined (ND) for the related pathogen.

#### Sample size calculation and analysis

Using a standard approach for calculating sample size for cluster Randomised Controlled Trials [30] we estimated the minimum detectable difference in primary and secondary health outcome measures with a planned total sample size of 750 children (375 intervention, 375 controls) across 50 clusters (25control/25 intervention) and with an anticipated intra-class correlation co-efficient (ICC) of 0.01. Our assumptions regarding baseline/control prevalence of any enteric infection and diarrhoeal disease are drawn from the most recent Multiple Indicator Cluster Survey (MICS) estimates for the prevalence of stunting and recent diarrhoea in Nyanza province [18], and the Demographic and Health Surveillance (DHS) survey national urban estimates for Kenya [31]; alongside, the national (Kenyan) and global estimates for prevalence of any enteric infection from the Global Enteric Multi-country Study (GEMS) [19]. In the absence of published effect size estimates for similar early childhood interventions on enteric infection prevalence and our assumption regarding effect size is cautiously estimated based on the effects on diarrhoea of different WASH interventions [32].

For the primary outcome, with 750 infants enrolled, and assuming a control prevalence of ≥1 of the 23 measured enteric infections of 0.7, and an intraclass correlation coefficient (ICC) of 0.01 we would have 80% power at a 5% level of significance to detect a minimum difference between arms in the prevalence of ≥1 infection of 11%. For our secondary outcome, with 750 infants, we would be able to detect a minimum difference in longitudinal prevalence of caregiver reported diarrhoea of 7% or greater, assuming a control longitudinal prevalence of diarrhoea of 15%.

The CONSORT Statement for cluster randomised controlled trials will guide the analysis and presentation of results [33]. To assess any imbalance between arms, descriptive statistics of demographic and outcome measures (where available) will be tabulated at baseline.

All analysis will be carried out on groups as randomised (‘intention to treat’). All analyses will account for the nature of the distribution of the relevant outcome and results will be presented as appropriate effects sizes at 95% confidence intervals. We account for clustering by using generalised estimating equations (GEE) and adjust for baseline differences in groups by including the cluster mean of our outcome at baseline as a covariate in statistical models. For all analyses, unadjusted and adjusted results will be presented, with covariates in adjusted analyses specified a priori.

#### Randomisation

Randomisation was undertaken remotely by the Clinical Trials Unit at the London School of Hygiene & Tropical Medicine (LSHTM). The unit of randomisation is the CHV catchment cluster, and, in discussion with the Ministry of Health for Kisumu County, the participating 50 clusters were selected from the 94 eligible clusters in the study neighbourhoods, with eligibility determined by the presence of an “active” CHV. The 50 active clusters were then randomly allocated 1:1 into two trial arms.

#### Blinding

This is a public health intervention seeking to change specific behaviours through direct engagement with participants such that blinding of participants to their allocation was not deemed possible. Randomisation of clusters was done remotely; enumerators, principal investigator, and trial statistician were blinded to allocation. The trial statistician will conduct final analyses blind to allocation.

#### Coordinating committees

The Trial Management Group includes representatives from each partner organisation (GLUK, Iowa University and LSHTM) chaired by the Principal Investigators (JM and OC). Modifications required to the protocol (intervention, participants, study design, analysis methods, or outcomes) during the study will be approved by the LSHTM Research Ethics Committee prior to implementation and the new information registered on the trial registry (clinicaltrials.gov). Need and frequency of audits for trials is independent of the investigators and is determined using a risk-based approach.

#### Adverse events

The trial is monitored for adverse events and all reported adverse events are documented and reports are compiled on a quarterly basis. The principal investigators (JM and OC) will review any reported severe adverse events to assess the level of relatedness to intervention and take appropriate action.

### Limitations

We had initially intended for the Safe Start intervention to be delivered exclusively by CHVs to demonstrate more directly the scalability of such an intervention within the existing health system structure and resource envelope. However, findings from our formative work demonstrated that such an approach would likely place undue burden on CHVs in the context of a research project. Although delivered by specialized field workers employed for the purposes of this study, our intervention is still considered to be deliverable within the CHV system and has been endorsed as such by the Ministry of Health for Kisumu County.

## Discussion

The goal of the ‘Safe Start’ intervention is to demonstrate that low cost, locally appropriate food hygiene interventions which target child caregivers of weaning infants can reduce foodborne exposure to enteric pathogens and the resulting infection and disease. Our intervention, informed by extensive formative research with infants, caregivers, health extension workers and discussion with the local Ministry of Health, has the potential to be scaled up if proven to be effective.

### Trial status

Protocol version number and date: Version 1, March 01, 2018.

Date recruitment began: March 26th, 2018.

Approximate date when study will be completed: November 30th, 2019.

## Supplementary information

**Additional file 1.** Intervention materials, the “Successful Girl” calendar.

## Data Availability

Not applicable.

## Abbreviations

CFU: Colony forming units
CHC: Community Health Committee
CHEW: Community Health Extension Worker
CHV: Community Health Volunteer
CLTS: Community Led Total Sanitation
cRCT: Cluster Randomized Control Trial
CU: Community Unit
DHS: Demographic Health Survey
GEMS: Global Enteric Multicenter Study
GLUK: Great Lakes University of Kisumu
GPS: Global Positioning System
ICC: Intraclass Correlation Coefficient
ICH-GCP: International Council for Harmonisation – Good Clinical Practice
LMIC: Low and Middle Income Countries
LSHTM: London School of Hygiene and Tropical Medicine
MICS: Multiple Indicator Cluster Survey
MSD: Moderate and Severe Diarrhoea
ORT: *Oral rehydration therapy* (*ORT*)
TIPS: Trial of Improved Practice
WASH: Water Sanitation and Hygiene
