# Supplementary figures and images for: The Safe Start trial to assess the effect of an infant hygiene intervention on enteric infections and diarrhoea in low-income informal neighbourhoods of Kisumu, Kenya: a study protocol for a cluster randomized controlled trial
Source: BMC Infect Dis. 2019 Dec 19;19:1066. doi: 10.1186/s12879-019-4657-0 (PMC6923833; doi:10.1186/s12879-019-4657-0)

**Additional file 1**:Intervention materials, the “Successful Girl” calendar


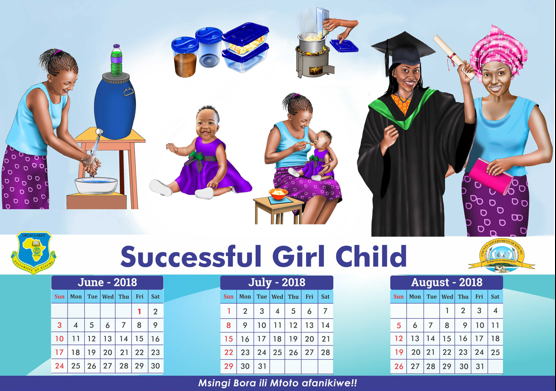

Supplement: Supplementary file 1 — Additional file 1. Intervention materials, the “Successful Girl” calendar. [file 12879_2019_4657_MOESM1_ESM.docx]
